# Changes in Cortisol Awakening Response During 10 Days of High-Intensity Cycling Exercise

**DOI:** 10.3390/life15121872

**Published:** 2025-12-06

**Authors:** Yui Ogasawara, Takayuki Sugo, Hironobu Tsuchiya

**Affiliations:** 1 Faculty of Liberal Arts, Sciences and Global Education, Osaka Metropolitan University, 2-1-132 Morinomiya, Joto-ku, Osaka 536-8525, Japan; 2 Department of Sport Sciences, Osaka University of Health and Sport Sciences, 1-1 Asashirodai, Kumatori-cho, Sennan-gun, Osaka 590-0406, Japan

**Keywords:** biomarker, endocrine response, physiological adaptation

## Abstract

Previous studies suggest that the cortisol awakening response (CAR) shows a biphasic pattern—either an increase or a blunting—in response to exercise involving overload, potentially reflecting physiological adaptation. However, its response to continuous high-intensity exercise under controlled experimental conditions has not been sufficiently investigated. This pilot case series examined daily CAR changes during a 10-day high-intensity cycling protocol (20 min/day at 80% of maximal oxygen uptake [$\overset{˙}{V}O_{2}\max$]) in two healthy male participants. The CAR increased during Days 1–4 and returned to baseline levels from Day 5 onward, showing similar trends in acute physiological responses. $\overset{˙}{V}O_{2}\max$ and/or maximum workload improved following the intervention. These findings support the methodological feasibility of longitudinal CAR monitoring during short-term high-intensity exercise under controlled experimental conditions and suggest that CAR may be a promising non-invasive biomarker for assessing short-term physiological adaptation.

## 1. Introduction

Exercise improves physical performance through metabolic, morphological, and neuromuscular adaptations [1,2,3,4,5]. However, when training loads exceed an individual’s adaptive capacity and recovery is insufficient, performance may decline, increasing the risk of overtraining [6]. To detect and prevent overtraining and optimize training adaptation, various biomarkers have been investigated to monitor physiological adaptation to exercise [7,8].

Among these biomarkers, cortisol—a final product of the hypothalamic–pituitary–adrenal (HPA) axis—is widely studied because its secretion increases in response to exercise as a stressor, depending on the intensity [9,10], duration [11], and volume of training [12,13]. Cortisol exhibits a well-established diurnal rhythm, characterized by a peak in the early morning and a gradual decline throughout the day [14,15,16]. A pronounced increase in cortisol levels typically occurs within the first 30 min after awakening, known as the cortisol awakening response (CAR) [17]. The CAR has demonstrated intra-individual reliability across multiple days [17] and can be measured noninvasively via saliva sampling. When methodological confounders are adequately controlled, the CAR exhibits a robust and consistent biphasic pattern, characterized by either an increase or a blunting, which is considered to potentially reflect the severity of stress-related symptoms in individual adaptation [18]. Therefore, the CAR has been widely studied as a biomarker of individual stress-related adaptation, particularly in the field of psychoneuroendocrinology [18].

Recently, the CAR has gained attention as a potential marker for monitoring physiological adaptation to exercise [19]. However, studies examining exercise-induced changes in the CAR remain limited, particularly under controlled laboratory conditions. Two studies have begun to address this gap: Ogasawara et al. [20] examined the effects of 20-min cycling at 40%, 60%, and 80% of maximal oxygen uptake ($\overset{˙}{V}O_{2}\max$) on the CAR, reporting elevated CAR levels the following morning under the 80% condition compared to rest. In contrast, Anderson et al. [21] found that 1-h cycling at 70–75% peak power output in a hot and humid environment resulted in a low CAR. These laboratory-based findings suggest a biphasic pattern in CAR changes following acute high-intensity exercise, potentially reflecting the impact of exercise load from the previous day.

To our knowledge, no studies have investigated CAR changes in response to repeated, laboratory-controlled exercise stimuli administered over consecutive days. Anderson and Wideman [19] speculated that the CAR may increase during the initial phase of training and stabilize as physiological adaptation progresses. Minetto et al. [22] conducted a field-based study in soccer players and reported that CAR increased in some individuals and decreased in others before and after a 7-day intensive training period, although training volume was not quantified and daily CAR fluctuations were not assessed. Considering that training adaptation is believed to result from the cumulative effects of repeated exercise stimuli [23], it is important to further investigate CAR changes using structured and continuous exercise protocols under controlled conditions.

Therefore, this pilot case series aimed to explore the feasibility of longitudinal CAR monitoring during short-term high-intensity exercise under controlled laboratory conditions. Spina et al. [24] reported increased mitochondrial enzyme activity and peak oxygen uptake ($VO_{2}\mathrm{peak}$) following 7–10 days of cycling at 60–70% $VO_{2}\mathrm{peak}$. Based on this finding, we hypothesized that 10 consecutive days of cycling at 80% $\overset{˙}{V}O_{2}\max$—a condition previously shown to elevate CAR [20]—would induce CAR changes associated with training adaptation. While the exact timing of adaptation within the 10-day period remains unclear, we adopted the hypothesis proposed by Anderson and Wideman [19]: that CAR increases during the initial phase of training and stabilizes as adaptation occurs.

Importantly, this study does not aim to generalize physiological responses, but rather to explore the methodological feasibility of longitudinal CAR monitoring in a controlled setting. Although the small sample size and absence of a comparison condition limit causal inference and generalizability, by observing changes from baseline within subjects, preliminary insights were obtained into the temporal dynamics of CAR in response to repeated exercise stimuli.

## 2. Materials and Methods

### 2.1. Participants

Four healthy adult males participated in this study. None of the participants had a history of smoking, irregular sleep patterns, hormonal disorders, psychiatric conditions, chronic low-carbohydrate diets, or habitual use of anabolic steroids and non-steroidal anti-inflammatory drugs. Given the approximately two-week duration of the experimental protocol and the substantial time commitment required, a convenience sampling method was employed. Four participants were selected based on their availability, willingness, and ability to complete the 10 consecutive days of high-intensity cycling exercise sessions required in the study.

However, one participant was excluded from the analysis due to concerns that his data may have been influenced by a swimming competition he had participated in two days prior to the start of the experiment. Another participant was also excluded because his CAR values were consistently unstable throughout the experimental period, raising concerns about potential HPA axis dysfunction unrelated to the study. Consequently, data from two participants were included in the final analysis and presented as individual cases (Participant A: age 22 years, height 170 cm, body weight 60.6 kg; Participant B: age 22 years, height 175.9 cm, body weight 65.6 kg).

Although the use of convenience sampling and the reporting of individual cases limit the generalizability of the findings, the data obtained provide valuable preliminary insights that may inform future research in this specialized area.

Prior to the experiment, all participants provided both verbal and written informed consent. This study was conducted in accordance with the Declaration of Helsinki and approved by the Ethics Committee of Osaka University of Health and Sport Sciences (approval number: 20-4).

### 2.2. Study Design and Procedures

Participants visited the laboratory a total of 14 times during the study period. On the first visit, $\overset{˙}{V}O_{2}\max$ was assessed using a maximal graded exercise test on a cycle ergometer. The test began at 0 W with a cadence of 60 revolutions per minute (rpm) and increased by 30 W every 2 min until the participants reached volitional exhaustion. Throughout the test, respiratory gases and heart rate (HR) were continuously measured, and ratings of perceived exertion (RPE) were recorded at the end of each exercise stage using the Borg 6–20 scale [25]. $\overset{˙}{V}O_{2}\max$ was considered valid and reliable based on the criteria outlined in the previous study [10], if at least three of the following four conditions were met: (1) an increase in $\overset{˙}{V}O_{2}$ of ≤0.15 L/min with increasing workload, (2) HR within ±5% of the age-predicted maximum, (3) respiratory exchange ratio (RER) ≥ 1.1, and (4) RPE ≥ 18.

Between 2 and 7 days after the $\overset{˙}{V}O_{2}\max$ assessment, participants engaged 12 consecutive days of experimental sessions. The first two days consisted of seated rest sessions lasting 20 min, serving as baseline measurements. The subsequent 10 days involved 20-min cycling exercise sessions performed daily at the fixed workload that was set on the first day, corresponding to 80% of each participant’s $\overset{˙}{V}O_{2}\max$. The session protocol was based on our previous study [20].

To minimize intra- and inter-individual biological variation, all sessions were conducted at a standardized start time of 18:00 ± 30 min. During the rest sessions, participants were allowed to engage in light conversation and use smartphones, but sleep was prohibited. During the cycling exercise sessions, HR and respiratory gases were continuously monitored, and RPE were recorded every 10 min. Saliva samples were collected at five points: before the session (Pre), immediately after (Post-0), and at 10, 20, and 30 min post-exercise (Post-10, Post-20, Post-30). After each session, participants consumed a commercially prepared bento meal for dinner and received instructions regarding the nighttime and next-morning measurements. They were also provided with saliva collection tubes and straws before returning home.

At home, participants collected saliva samples at 21:00 (recovery 1) and 23:00 (recovery 2). Reminder messages were sent 15 min prior to each collection, and participants confirmed successful sampling. After recovery 2, participants received instructions for the next-morning measurements and a URL link to a Google Form was sent to participants for recording contextual information related to the next morning’s saliva sampling.

Upon waking the next morning, participants collected saliva samples at three points: immediately upon waking (*C*_0_), 15 min after waking (*C*_15_), and 30 min after waking (*C*_30_), for the evaluation of CAR. Between waking and 30 min post-waking, participants were instructed to complete a Google Form to record contextual information, including bedtime, wake time, saliva sampling times, and any relevant events during sleep or upon waking. The form automatically recorded a submission timestamp, which was used to verify adherence to the sampling time. They were asked to submit the form immediately after completing the *C*_30_ saliva sample. If discrepancies were identified between the reported *C*_30_ sampling time and the form submission time, participants were contacted to verify the accuracy of the morning procedures. Although specific bedtimes and wake times were not prescribed, participants were instructed to maintain similar sleep and wake schedules across the 12-day period. Participant A had an average bedtime of 1:31 ± 1.0 h, wake time of 7:06 ± 1.2 h, and sleep duration of 5.9 ± 1.2 h across the 12 days. Participant B had an average bedtime of 1:19 ± 0.4 h, wake time of 7:47 ± 0.7 h, and sleep duration of 6.5 ± 0.6 h. Saliva samples collected at night and in the morning were stored in a household refrigerator (4 °C) and submitted to the researchers at the next session.

On the day following the final (Day 10) cycling exercise session, a second $\overset{˙}{V}O_{2}\max$ test was conducted using the same protocol and time of day as the initial assessment.

To minimize, as much as possible, the influence of confounding factors unrelated to the experimental exercise, participants were instructed to abstain from engaging in any strenuous physical activity, as well as from consuming caffeine and alcohol, from the day prior to the start of the study until its completion. In addition to standardizing dinner, participants were asked to maintain a consistent dietary pattern throughout the study period. Furthermore, the intake of food and carbohydrate-containing beverages was prohibited starting 4 h before each session and again from 20:00 after the session until the completion of the following morning’s measurements. After returning home, participants were prohibited from engaging in part-time work and from drinking water, bathing, or brushing their teeth starting 1 h prior to saliva sampling. During the morning sampling period, participants were instructed to refrain from brushing their teeth, drinking water, bathing, or returning to sleep for 30 min after waking. However, routine activities such as changing clothes or using the toilet were permitted, provided they did not significantly affect arousal levels. Exposure to environmental light upon waking was not restricted, insofar as it was necessary for the performance of these activities.

### 2.3. Saliva Sample

Saliva samples were collected using the passive drool method, in which participants allowed saliva to accumulate naturally in the oral cavity for 2 min and then transferred it into a collection tube via a straw. The collected samples were centrifuged at 3000 rpm for 5 min and stored at −80 °C until analysis. On the day of analysis, samples were thawed, and cortisol concentrations were determined using an enzyme-linked immunosorbent assay (ELISA) with a commercially available kit (Cortisol ELISA Kit [RE52611], IBL International GmbH, Hamburg, Germany).

### 2.4. Statistical Analysis

As this study reports individual data, no statistical comparisons were performed. Instead, descriptive statistics were used to present each participant’s data, including mean values, standard deviations, and daily measurements for each variable. In addition, effect sizes (Hedges’ g) and their 95% confidence intervals (CI) were calculated to provide a standardized measure of change. Effect size interpretation followed conventional thresholds: small (0.2), medium (0.5), and large (0.8). Given the limited number of data points within each period, confidence intervals are presented as reference values, and caution should be exercised when interpreting their precision.

Given the exploratory nature of this pilot case series and the small sample size (*n* = 2), statistical comparisons were not appropriate. In case reports, descriptive statistics and visual inspection are commonly employed to identify trends rather than formal hypothesis testing [26]. This approach aligns with the methodological aim of assessing the feasibility of longitudinal CAR monitoring under controlled exercise conditions.

Due to the extended commitment required during the approximately two-week duration of the study, no familiarization period was implemented. As a result, both participants exhibited elevated cortisol levels on the first day of baseline measurement, likely reflecting psychological stress or lack of adaptation to the experimental setting. Therefore, cortisol data from the first rest session were excluded from subsequent analyses, as they could not be considered valid baseline values. This limitation should be acknowledged when interpreting the findings of the present study.

## 3. Results

### 3.1. Physiological and Perceptual Responses

Table 1 presents the data for HR, $\overset{˙}{V}O_{2}$, and RPE during the 20-min cycling exercise sessions over 10 consecutive days for Participants A and B.

For Participant A, HR showed a very large negative effect size when comparing Days 1–4 and Days 5–7 at 10 min (*g* = −2.13, *95% CI* [−4.13, −0.13]) and a similarly large negative effect at 20 min (*g* = −1.93, *95% CI* [−3.85, −0.01]), indicating a tendency for HR to decrease during the mid-phase of the cycling exercise session. When comparing Days 1–4 and Days 8–10, the decrease was even more pronounced, with a very large negative effect at 10 min (*g* = −2.88, *95% CI* [−5.22, −0.55]) and an even larger negative effect at 20 min (*g* = −3.18, *95% CI* [−5.66, −0.70]), suggesting a further decline in the late-phase of the cycling exercise session. For $\overset{˙}{V}O_{2}$, Days 1–4 versus Days 5–7 showed a small negative effect at 10 min (*g* = −0.08, *95% CI* [−1.34, 1.18]) and a moderate negative effect at 20 min (*g* = −0.81, *95% CI* [−2.14, 0.52]). Days 1–4 versus Days 8–10 showed a small effect at 10 min (*g* = 0.08, *95% CI* [−1.18, 1.34]) and a moderate negative effect at 20 min (*g* = −0.58, *95% CI* [−1.88, 0.71]), indicating a tendency for $\overset{˙}{V}O_{2}$ to decline from mid- to late-phase of the cycling exercise session. For RPE, Days 1–4 versus Days 5–7 showed a small negative effect at 10 min (*g* = −0.38, *95% CI* [−1.90, 1.13]) and a moderate negative effect at 20 min (*g* = −0.67, *95% CI* [−2.22, 0.89]), suggesting a tendency for perceived exertion to decrease during the mid-phase of the cycling exercise session. Days 1–4 versus Days 8–10 showed an effect size near zero at 10 min (*g* = 0.00, *95% CI* [−1.50, 1.50]) and a small negative effect at 20 min (*g* = −0.49, *95% CI* [−2.01, 1.04]), indicating only minor changes in the late-phase.

For Participant B, HR showed a large negative effect when comparing Days 1–4 and Days 5–7 at 10 min (*g* = −1.32, *95% CI* [−3.02, 0.39]) and at 20 min (*g* = −1.48, *95% CI* [−3.24, 0.28]), indicating a tendency for HR to decrease during the mid-phase of the cycling exercise session. Comparing Days 1–4 and Days 8–10 revealed a very large negative effect at 10 min (*g* = −2.13, *95% CI* [−4.13, −0.13]) and an even larger negative effect at 20 min (*g* = −3.34, *95% CI* [−5.90, −0.78]), suggesting a further decline in the late-phase of the cycling exercise session. For $\overset{˙}{V}O_{2}$, Days 1–4 versus Days 5–7 showed a very large negative effect at 10 min (*g* = −2.46, *95% CI* [−4.26, −0.66]) and an even larger negative effect at 20 min (*g* = −3.53, *95% CI* [−5.76, −1.29]). Days 1–4 versus Days 8–10 showed similarly large effects at 10 min (*g* = −2.48, *95% CI* [−4.29, −0.67]) and 20 min (*g* = −3.89, *95% CI* [−6.29, −1.49]), indicating a marked decline in $\overset{˙}{V}O_{2}$ from mid- to late-phase of the cycling exercise session. For RPE, Days 1–4 versus Days 5–7 showed a large negative effect at 10 min (*g* = −1.63, *95% CI* [−3.44, 0.18]) and at 20 min (*g* = −1.71, *95% CI* [−3.55, 0.12]), suggesting a tendency for perceived exertion to decrease during the mid-phase of the cycling exercise session. Days 1–4 versus Days 8–10 showed a very large negative effect at 10 min (*g* = −3.81, *95% CI* [−6.61, −1.00]) and at 20 min (*g* = −3.30, *95% CI* [−5.83, −0.76]), indicating a further decline in the late-phase of the cycling exercise session.

In summary, all variables showed the highest values during Days 1–4, with a tendency for reduced responses observed in Days 5–7 and Days 8–10.

### 3.2. Acute Cortisol Responses

**Figure 1:**
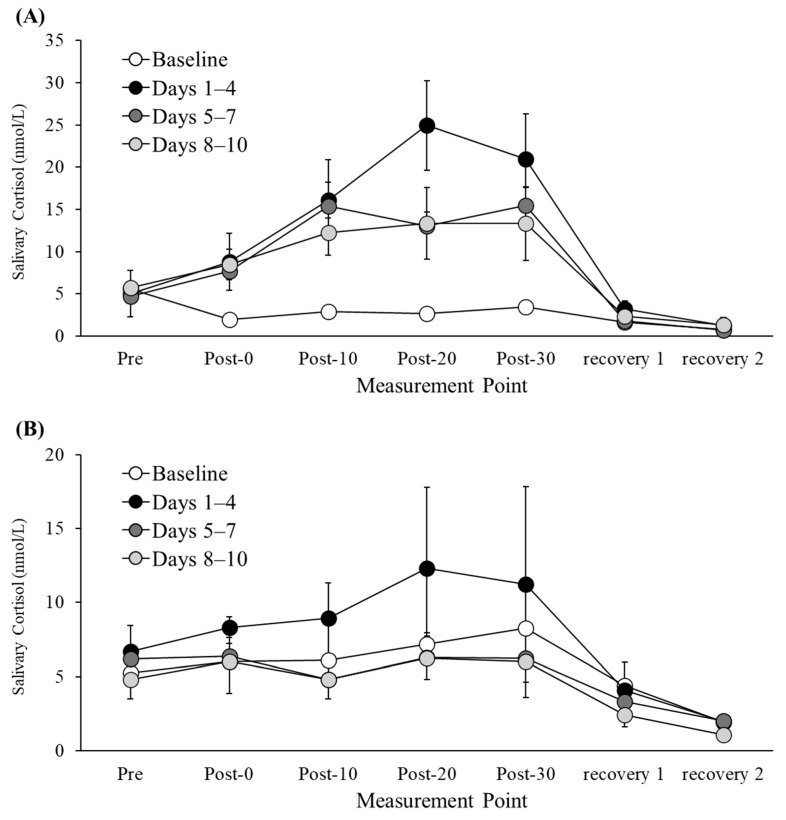
Changes in acute cortisol concentrations during the experimental session period for Participant A (**A**) and Participant B (**B**). Values represent the second-day rest session and the means ± SD for the cycling exercise sessions Days 1–4, 5–7, and 8–10, respectively.

Figure 1 illustrates the temporal changes in acute salivary cortisol concentrations for Participants A and B throughout the experimental session period.

For Participant A, in the comparison between Days 1–4 and Days 5–7, a small negative effect size was observed at Post-0 (*g* = −0.29, *95% CI* [−1.80, 1.22]), indicating a slight tendency for salivary cortisol concentration to decrease. At Post-10, the decrease was mild (*g* = −0.13, *95% CI* [−1.63, 1.37]), whereas at Post-20, a very large negative effect size was found (*g* = −2.03, *95% CI* [−3.99, −0.07]), suggesting a marked reduction. At Post-30, a large negative effect size persisted (*g* = −0.92, *95% CI* [−2.53, 0.68]), indicating that the decreasing trend continued. In the comparison between Days 1–4 and Days 8–10, the effect size at Post-0 was nearly zero (*g* = −0.08, *95% CI* [−1.58, 1.42]), showing minimal change. At Post-10, a large negative effect size was observed (*g* = −1.16, *95% CI* [−2.82, 0.50]), indicating a clear tendency toward reduction. At Post-20, an even larger negative effect size was found (*g* = −1.69, *95% CI* [−3.52, 0.14]), confirming a pronounced decrease. At Post-30, a large negative effect size remained (*g* = −1.10, *95% CI* [−2.75, 0.54]), suggesting that the downward trend persisted.

For Participant B, in the comparison between Days 1–4 and Days 5–7, a very large negative effect size was observed at Post-0 (*g* = −1.78, *95% CI* [−3.65, 0.08]), indicating a substantial decrease in salivary cortisol concentration. At Post-10, a large negative effect size was maintained (*g* = −1.60, *95% CI* [−3.40, 0.20]), showing continued reduction. At Post-20, the negative effect size diminished somewhat (*g* = −0.99, *95% CI* [−2.62, 0.63]), suggesting that the decrease persisted but was less pronounced. At Post-30, the effect size further decreased (*g* = −0.69, *95% CI* [−2.25, 0.86]), indicating a weakening of the downward trend.

In the comparison between Days 1–4 and Days 8–10, a large negative effect size was observed at Post-0 (*g* = −1.07, *95% CI* [−2.71, 0.57]), indicating a tendency toward reduction. At Post-10, an even larger negative effect size was found (*g* = −1.46, *95% CI* [−3.21, 0.29]), suggesting a stronger decrease. At Post-20, a similar negative effect size was maintained (*g* = −1.01, *95% CI* [−2.63, 0.61]), indicating that the reduction persisted. At Post-30, the effect size slightly decreased (*g* = −0.71, *95% CI* [−2.27, 0.85]), suggesting a weakening of the downward trend.

To summarize, similar to the patterns observed in HR, $\overset{˙}{V}O_{2}$, and RPE data, the highest acute salivary cortisol responses were recorded during Days 1–4 (early phase of the cycling exercise session), with a tendency for decreased responses during Days 5–7 (mid-phase) and Days 8–10 (late-phase).

### 3.3. CAR Data

**Figure 2:**
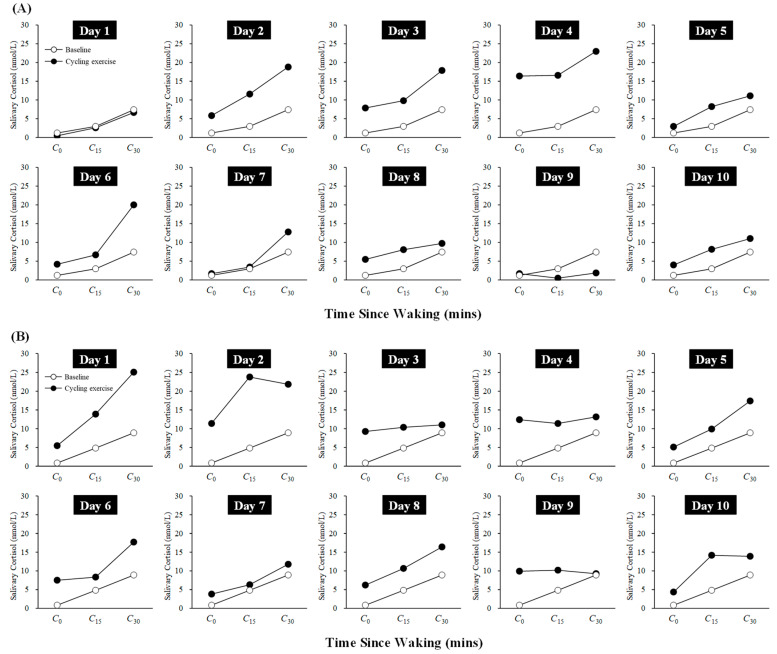
Changes in CAR during the 10-day cycling exercise session period for Participant A (**A**) and Participant B (**B**). Baseline values represent the second-day rest session and are included in each figure for comparison with the values obtained during the 10-day cycling exercise sessions.

Figure 2 shows the changes in CAR from baseline for Participants A and B during the 10-day cycling exercise session period. Participant A exhibited an increase in CAR during Days 1–4, followed by a tendency to return to baseline levels from Day 5 onward. Participant B showed a rise in CAR during Days 1–2, with a similar tendency to return to baseline levels from Day 3 onward. Taken together, these results suggest that both participants experienced a transient increase in CAR during the initial phase of the cycling exercise sessions (Days 1–4).

### 3.4. Aerobic Capacity and Work Performance

Table 2 presents the $\overset{˙}{V}O_{2}\max$ and maximal workload values for Participants A and B before and after the 12-day experimental session intervention. Participant A showed a 5.87% increase in $\overset{˙}{V}O_{2}\max$, along with a 30 W improvement in maximal workload. Participant B exhibited a 3.3% decrease in $\overset{˙}{V}O_{2}\max$, but also demonstrated a 30 W increase in maximal workload. In summary, both participants showed improvements in aerobic capacity and/or work performance following the intervention.

## 4. Discussion

The purpose of this pilot case series was to explore the feasibility of longitudinal CAR monitoring during a 10-day period of consecutive high-intensity cycling exercise at the fixed workload under controlled laboratory conditions. The main findings of this study were: (1) acute responses in HR, $\overset{˙}{V}O_{2}$, RPE, and cortisol were highest during the initial phase of the cycling sessions (Days 1–4), with subsequent reductions observed thereafter; (2) CAR showed a transient increase during Days 1–4, followed by a return to baseline levels; and (3) both participants demonstrated improvements in maximal oxygen uptake and/or maximal workload following the intervention. To the best of our knowledge, this is the first study to experimentally demonstrate changes in CAR in response to short-term consecutive exercise sessions.

The acute responses in HR, $\overset{˙}{V}O_{2}$, RPE, and cortisol measured on each session were consistently highest during the initial phase of the cycling exercise sessions for both participants, followed by a decreasing trend thereafter. These results are consistent with previous studies. Spina et al. [24] reported that 7–10 days of cycling exercise increased mitochondrial enzyme activity in skeletal muscle, leading to a glycogen-sparing effect during exercise at the same workload, which in turn resulted in reductions in RER and HR. Additionally, Viru [27] suggested that endurance training elevates the exercise intensity threshold required to elicit an acute cortisol response, potentially reducing or eliminating such responses to exercise at the same workload. Furthermore, McMurray and Hackney [28] reported that acute cortisol responses to exercise are elicited in proportion to the metabolic demands of the exercise performed. Based on these findings, it is plausible that the 10-day high-intensity cycling intervention in the present study enhanced the participants’ endurance capacity and improved the efficiency of energy substrate utilization, thereby reducing the metabolic demands of exercise at the fixed workload. As a result, the relative exercise workload imposed by the fixed workload may have progressively decreased relative to the initial session, thereby leading to the reductions in acute physiological responses observed from Day 5 onward.

Focusing on changes in CAR, the results exhibited a trend consistent with the proposed hypothesis, as both participants showed a transient increase on Days 1–4, followed by a return to near baseline levels. The elevation in CAR observed during the early phase of the intervention is consistent with our previous findings [20] and with other studies [22], and may indicate a transient activation of the HPA axis in response to the onset of high-intensity exercise. Moreover, the recovery of CAR around Day 5 aligns with the inference made by Anderson and Wideman [19] in their review, indicating that physiological adaptation to the imposed exercise may have led to a reduction in HPA axis reactivity. The occurrence of adaptation around Day 5 is further supported by the observed trends in acute physiological responses and the improvements in $\overset{˙}{V}O_{2}\max$ and/or maximal workload following the cycling exercise intervention, which are also consistent with the approximately 9% increase in $VO_{2}\mathrm{peak}$ reported by Spina et al. [24].

On the other hand, the transient increase and subsequent normalization of the CAR may also reflect initial disruptions associated with participation in the experiment and subsequent habituation to the experimental environment. The CAR has been shown to be influenced by a variety of state covariates, including wake-up time, sleep duration and quality, experiences from the previous day, and expectations for the upcoming day [29]. In both participants of this case series, elevated cortisol levels were observed on the first day of baseline measurement, likely due to circumstances suggesting insufficient adaptation to the experimental environment. To account for these factors, participants were asked to report events during sleep and upon awakening via a Google Form, and were also instructed to describe any psychological or physical burdens associated with study participation. Based on these reports, it is reasonable to assume that a certain degree of control was achieved from the second day of baseline measurement onward. Additionally, considering that interventions such as nutrition, stretching, and hydrotherapy can influence post-exercise recovery [30], participants’ behaviors during the experimental sessions—such as sleep quality, dietary intake beyond the prescribed meals, physical activity levels, stretching routines, and bathing practices—may have affected both the magnitude of CAR elevation and the facilitation and/or delay of recovery toward baseline values. However, given that this study did not comprehensively assess other physiological or psychological indicators related to these factors, and their potential influence cannot be completely ruled out. Moreover, two participants who were excluded from the study showed data that raised concerns about potential effects of experiences unrelated to the experiment in the days prior. Considering these points, future research should incorporate additional measures to more comprehensively evaluate participants’ contextual conditions.

Furthermore, cortisol exhibits pronounced diurnal and intra-individual variability [31]. The CAR is regarded as a relatively distinct feature within circadian rhythmicity and is influenced by factors such as awakening time and light exposure [29]. In this case series, we verified compliance with sampling times using time-stamped Google Forms and instructed participants to maintain similar bedtimes and wake times over 12 consecutive days; however, exact times and environmental light exposure were not strictly controlled. These procedures likely allowed us to control these factors to some extent, although the influence of specific state-related variables may remain. Our findings primarily indicate changes in the CAR in response to continuous high-intensity exercise, but careful interpretation is warranted considering these potential influences.

The primary significance of this study lies in demonstrating a tendency consistent with the inferential claim made by Anderson and Wideman [19], regarding changes in the CAR following continuous high-intensity exercise. This finding supports the methodological validity of longitudinal monitoring of the CAR in response to controlled continuous exercise stimuli. These findings also suggest that CAR may have the potential to serve as a useful non-invasive biomarker for monitoring short-term physiological adaptations to high-intensity exercise. However, many aspects of how exercise influences the CAR remain unclear. Although previous studies [20,21,32] and the present study have controlled and quantified exercise conditions and training volume, few physiological indicators beyond salivary and blood cortisol concentrations have been assessed to evaluate the participants’ physical state. Therefore, it is currently difficult to determine precisely what physiological conditions are reflected by exercise-induced changes in the CAR. According to a review by Stalder et al. [33], the CAR is thought to contribute to the preparation of the physiological system for daytime activity, potentially involving the synchronization of peripheral clocks related to energy metabolism, immune function, and neurocognitive systems upon awakening. Additionally, previous studies have reported that physiological responses such as reduced muscle glycogen content and muscle damage can persist into the days following high-load exercise [34,35]. Taken together, these findings suggest that exercise-induced changes in the CAR may play a regulatory role in response to physiological disturbances in peripheral tissues caused by exercise. Future research should incorporate complementary physiological markers, such as glucose and inflammatory cytokines, to comprehensively assess the peripheral physiological states associated with changes in the CAR.

The primary limitation of this study is its case-series design, which included only two healthy young male participants, thereby restricting the generalizability of the findings. Cortisol responses to exercise are important for tissue growth and remodeling [36] and are known to be modulated by habitual training [37]. Moreover, sex differences in physiological responses to exercise have also been reported [38]. These factors may influence variations in the CAR in response to exercise; therefore, future research should involve larger and more diverse samples that account for training background, fitness level, and sex to verify the reproducibility of these findings. Nevertheless, the current study employed a tightly controlled laboratory-based exercise protocol and implemented a 10-day consecutive intervention while minimizing the influence of extraneous variables. Although certain methodological challenges remain—such as the need for an adequate familiarization period prior to baseline assessments—the data obtained represents a valuable contribution to the field and provides preliminary insights that may inform future investigations.

## 5. Conclusions

This case series supports the methodological feasibility of longitudinal CAR monitoring during short-term high-intensity exercise under controlled experimental conditions and demonstrates the potential utility of the CAR as a non-invasive biomarker for short-term exercise-induced physiological adaptations. The initial elevations in physiological responses and the CAR likely reflect early activation of the HPA axis in response to the imposed exercise, while the subsequent reductions may indicate the progression of physiological adaptation. Beyond methodological implications, these findings may have practical relevance for training monitoring. The CAR could help coaches and practitioners optimize load progression and reduce maladaptation risk when introducing individuals to demanding exercise programs. Future research should aim to further clarify the physiological significance and generalizability of the CAR as a biomarker by incorporating a broader range of physiological indicators and larger, more diverse samples.

## Figures and Tables

**Table 1:** HR, $\overset{˙}{V}O_{2}$, and RPE responses during 20 min cycling exercise sessions over 10 Days.

	HR (bpm)			$\overset{˙}{V}O_{2}$ (mL/kg/min)			RPE		
	0 min	10 min	20 min	0 min	10 min	20min	0 min	10 min	20 min
Participant A									
Days 1–4	88.5 ± 3.0	167.8 ± 3.7	175.8 ± 4.3	10.5 ± 1.3	36.9 ± 1.1	40.6 ± 1.5	8.3 ± 0.8	15.0 ± 0.7	17.0 ± 1.2
Days 5–7	84.7 ± 2.4	159.3 ± 0.5	166.0 ± 2.4	10.1 ± 0.3	36.8 ± 1.7	39.1 ± 0.9	9.0 ± 0.0	14.7 ± 0.5	16.0 ± 0.8
Days 8–10	81.3 ± 2.4	154.0 ± 2.9	158.0 ± 3.6	9.7 ± 0.1	37.1 ± 2.0	38.9 ± 2.7	9.0 ± 0.0	15.0 ± 0.0	16.3 ± 0.5
Participant B									
Days 1–4	77.8 ± 0.8	161.3 ± 2.2	165.3 ± 1.8	8.1 ± 1.8	41.6 ± 0.6	42.5 ± 0.6	8.8 ± 0.8	15.8 ± 0.4	16.8 ± 0.4
Days 5–7	78.0 ± 4.3	157.3 ± 2.1	158.3 ± 4.6	9.8 ± 1.5	39.7 ± 0.4	39.8 ± 0.4	9.0 ± 0.0	15.0 ± 0.0	15.7 ± 0.5
Days 8–10	78.7 ± 6.0	155.3 ± 1.7	156.7 ± 1.9	7.7 ± 2.9	39.8 ± 0.3	39.8 ± 0.2	8.3 ± 0.5	14.0 ± 0.0	14.7 ± 0.5

Values represent the means ± SD for cycling exercise sessions Days 1–4, 5–7, and 8–10, respectively. HR, $\overset{˙}{V}O_{2}$, and RPE were measured at 0, 10, and 20 min during every cycling exercise session. HR data at 0, 10, and 20 min were selected from the continuous 20-min data. $\overset{˙}{V}O_{2}$ values at 0, 10, and 20 min were calculated as the average over the 1-min period before the start of the cycling exercise session, and over the intervals from 7 to 10 min and 17 to 20 min after the start, respectively.

**Table 2:** $\overset{˙}{V}O_{2}\max$ and maximum workload before and after the experimental session intervention.

	$\overset{˙}{V}O_{2}\max$ (mL/kg/min)	Maximum Workload (W)
Participant A Before After	44.3 46.9	270 300
Participant B Before After	51.0 49.3	300 330

## Data Availability

All data generated or analyzed during this study are included in this manuscript.
